# Eye, Ear, Nose and Throat

**Published:** 1903-04

**Authors:** 

**Affiliations:** Brownwood, Texas


### Eye, Ear, Nose and Throat.

EDITED BY DR. P. M. PAYNE, BROWNWOOD, TEXAS.

The *Laryngoscope* for July contains the obituaries of two of the world's most famous Laryngologists, Lenox Browne, F. R. C. S. E., and Dr. Morris Joseph Asch.

Lenox Browne was one of the founders of the "British Rhinological, Otological and Laryngological Association," and was one of its early presidents. He was also aural surgeon to the Royal Society of Musicians, surgeon to the Royal Choral Society, and consulting surgeon to the Newcastle Throat and Ear Hospital. He was author of the splendid work on "The Throat and Nose, and Their Diseases," so popular in the United States a few years ago.

Dr. Asch was, during the Civil War, surgeon in chief to the artillery reserve of the army of the Potomac; medical inspector of the army of the James, and staff surgeon to Gen. Phil. Sheridan, 1865-1873, but resigned from this last mentioned position to enter the practice of medicine in New York City. He was surgeon to the throat department of the New York Eye and Ear Infirmary and the Manhattan Eye and Ear Hospital.

Dr. Asch will live in the history of surgery for his invaluable operation on the nasal septum. The world at large recognizes the "Asch operation" as one of the permanent advances of modern surgery.

Chronic Inflammation of the Pharyngeal Tonsil without Interference With Nasal Breathing. Wm. R. Murry, Minneapolis.—*Northwestern Lancet,* Sept. 1, 1902.

Typical cases of adenoids, producing mouth breathing are fairly well recognized, but Murray calls attention to cases in which there is chronic inflammation with little or no hypertrophy, and offers the following conclusions: (1) That a chronic inflammation of the pharyngeal tonsil may exist without any obstruction to nasal respiration. (2) That many cases of post-nasal catarrh are due to the presence of a chronically inflamed pharyngeal tonsil. (3) That some cases of acute and chronic otitis media have their origin in an eustachian salpingitis, which was due to a low grade of inflammation of the pharyngeal tonsil. (4) That in the treatment of some cases of chronic otitis media the source of the trouble may be overlooked, and that no permanent improvement will follow until the underlying cause be removed. (5) That if the presence of a chronically inflamed pharyngeal tonsil were more generally recognized, and more thoroughly removed, there would be fewer cases of chronic otitis media.—*Laryngoscope,* Feb., 1903.

SELECTED ABSTRACTS FROM THE AMERICAN MED. ASSO. JOURNAL.

Davidson, in *Virginia Medical Semi Monthly,* advises the use of glasses, and thinks that the correction of nervous symptoms is an even more important indication for their use than the improvement of vision. The glass which accomplishes this is not always the one that gives the best distant vision from the field.

Sharp, according to an abstract in the *Indiana Medical Journal,* gives the following suggestions and directions for dropping solutions into the eye: Never apply the drops directly to the cornea, nor should the dropper be brought down in front of the eye, as the patient will close the eye reflexly. The dropper should be brought from below upward and to the outer canthus. Have the patient turn the eye upward and at the same time gently evert the lower lid, and while it is in that position place one or two drops on the conjunctiva of the everted lid—not more than two drops should be put in at one time, as the sac is not capable of handling more. The patient should be directed to look down, at the same time holding the upper lid until the lower one comes in contact with it. In this way none of the solution will drop on the cheek. Care should be taken that the dropper does not touch the eye anywhere, as it may infect the eye or in turn become infected by the eye and thus infect the medicine when replaced. It is well to boil the dropper before using it.

